# MyoRing Implantation with and without Corneal Collagen Crosslinking for the Management of Keratoconus

**DOI:** 10.18502/jovr.v15i4.7790

**Published:** 2020-10-25

**Authors:** Mehrdad Mohammadpour, Ahmad Masoumi, Mahmoud Dehghan, Mohammad Nasser Hashemian, Shahab Addin Karami, Alireza Mahmoudi

**Affiliations:** ^1^Translational Ophthalmology Research Center, Farabi Eye Hospital, Tehran University of Medical Sciences, Tehran, Iran

**Keywords:** Corneal Collagen Crosslinking, Keratoconus, MyoRing

## Abstract

**Purpose:**

To evaluate the safety and efficacy of femtosecond laser-assisted MyoRing implantation with concurrent corneal collagen crosslinking (CXL) compared to MyoRing alone for the treatment of progressive keratoconus.

**Methods:**

A total of 60 patients were enrolled in this randomized controlled trial. The patients were randomly allocated into two groups. In the first group, MyoRing was implanted, while in the second, it was inserted in the corneal stroma using the same technique, along with simultaneous CXL. Visual, refractive, topographic, and abberometric outcomes were measured preoperatively and at every postoperative visit.

**Results:**

Data of 47 patients were available at the end of the study; 28 in the MyoRing group and 19 in the MyoRing + CXL group. The mean uncorrected distance visual acuity (UDVA) improved from 0.79 ± 0.39 logMAR to 0.52 ± 0.31 logMAR (P < 0.05) in the MyoRing + CXL group and from 0.65 ± 0.38 logMAR to 0.62 ± 0.23 logMAR (P = 0.70) in the MyoRing group. CDVA changed from 0.33 ± 0.19 logMAR to 0.25 ± 0.16 logMAR (P = 0.10) in the MyoRing + CXL group and 0.32 ± 0.22 logMAR to 0.33 ± 0.17 logMAR (P > 0.50) in the MyoRing group. The mean keratometry (Km) decreased from 47.5 ± 2.7 D to 43.8 ± 3.2 D (P < 0.001) in the MyoRing group and 49.3 ± 3.4 D to 45.1 ± 3.0 D (P < 0.001) in the MyoRing + CXL group. Besides, horizontal coma was significantly lower in the MyoRing + CXL group (P = 0.022).

**Conclusion:**

MyoRing insertion combined with CXL is a safe and effective method for the treatment of keratoconus. The visual and topographic outcomes were comparable to that for MyoRing insertion after 10 months; however, horizontal coma was significantly lower in the MyoRing + CXL group.

##  INTRODUCTION

Keratoconus is a non-inflammatory corneal ectasia resulting in progressive thinning and steepening of the cornea. Patients usually present with decreased visual acuity, progressive myopia, irregular astigmatism, and central corneal scarring.^[1]^

A wide range of options is available for the treatment of keratoconus. In early stages, patients can be treated with spectacles or contact lenses. However, in advanced stages with corneal scarring, corneal transplantation is the only available option to restore vision. For patients without corneal scarring intolerant to contact lenses, the new modality, intracorneal ring segment (ICRS) or continuous intracorneal ring implantation in the corneal stroma may be used. The technique of insertion of a continuous intracorneal ring was developed in the late 20${}^{\mathrm{th}}$ century with the aim of correcting myopia. However, owing to technical difficulties and incision-related complications, continuous intracorneal rings were supplanted by ICRS, which had been used to correct mild to moderate myopia,^[2,3,4]^ but recently gained great popularity to treat keratoconus.^[5,6,7]^ Although ICRS cannot completely avert corneal transplantation, it can delay the keratoconus progression.^[8]^ ICRSs can flatten the corneal center and move the corneal apex to the center of cornea. It facilitates the fitting of the contact lens and optimizes the best corrected visual acuity.

The introduction of femtosecond laser technology in the field of refractive surgery offered new hopes to treat human refractive errors and also provided a new surgical modality to create tunnels for precise ICRS insertion. The femtosecond laser-assisted ICRS implantation has several advantages over mechanical approaches including a more uniform dissection, less patient discomfort, quick recovery, and more predictable results.^[9]^

Corneal collagen crosslinking (CXL) uses ultraviolet A (UVA) light and riboflavin to stiffen the corneal stroma. The irradiation of riboflavin results in formation of free radicals, inducing the formation of covalent bonds between the amino acid groups of collagen fibers.^[10,11]^ It halts the progression of keratoconus as shown by several studies.^[10,11][12][13]^ ICRS insertion can improve the corneal topography and correct myopia and astigmatism in keratoconic corneas; however, it has limited effect on the progression of keratoconus. In comparison, as stated earlier, CXL is a safe and effective method to stop the progression of ectatic corneal disorders. Thus, hypothetically, the benefits of both methods can be obtained by combining the two procedures. Several studies have been performed to test the safety and efficacy of the combined procedure.^[14,15,16,17]^ However, only few studies have evaluated same-day ICRS + CXL. This study was designed to assess and compare the long-term outcome of simultaneous insertion of MyoRing and CXL with MyoRing alone.

##  METHODS

This study was approved by the Institutional Review Board of Tehran University of Medical Sciences and was compliant with the tenets of the Declaration of Helsinki. Informed consent was obtained from all patients prior to the study. A total of 60 patients aged between 18 and 35 years who were diagnosed with keratoconus based on the clinical and Pentacam criteria were enrolled in this study. The inclusion criteria of the study were keratoconic eyes with central clear cornea, contact lens intolerance, and central corneal thickness > 380 µm. Patients who had previously undergone any ophthalmic surgery, pregnant and lactating women, and those with a history of collagen vascular diseases were excluded from this study. Patients were randomly allocated into two groups. In the first group, MyoRing was implanted at a depth of 300 µm using femtosecond laser. In the second group, MyoRing was implanted using the same technique with simultaneous CXL. Preoperative and postoperative examinations included uncorrected distance visual acuity (UDVA), corrected distance visual acuity (CDVA), dry and cycloplegic refraction, slit lamp biomicroscopy, dilated fundus examination, topographic and optical pachymetry using the Pentacam system (OCULUS, Wetzlar, Germany), and corneal abbermotry (iTrace, Tracey technologies, Houston, TX). Patients were examined one day before the surgery and one day, one month, three months, and ten months postoperatively. The information for this trial is available to the public through the Iranian National Registry for Clinical Trials (http://www.irct.ir).

### Surgical technique

All surgeries were done by the same experienced surgeon (MM) at Farabi Eye Hospital, Tehran, Iran. The procedure was performed under topical anesthesia using tetracaine 0.5% eye drops. The eyes were sterilized using povidone iodine, and an eyelid speculum was used to hold the eyes open. The cornea was marked in the pupillary center by a Sinskey hook as a reference point for pocket creation. A 300 μm deep tunnel was created in the corneal stroma using the femtosecond laser (Technolas 520F.........). The laser creates dissection planes by focusing a 3 μm diameter laser beam with a frequency of 500 kHz and a wavelength of 1054 nm at a predetermined stromal depth. A MyoRing with a diameter of 5 mm and a thickness of 240 µm was chosen for implantation. It was then inserted into the corneal pocket via a previously made temporal corneal incision. In the second group, after anesthetizing the eye and creating the pocket, riboflavin 0.1% was injected into the corneal pocket to completely fill it. After 5 min, diffusion of riboflavin was observed in the corneal stroma and anterior chamber. The MyoRing was then implanted using the mentioned technique. Finally, the cornea was exposed to 370-nm UVA light for 10 min with an irradiance of 9 mW/cm${}^{2}$. The MyoRing implantation and CXL procedure was uneventful without needing sutures.

Topical antibiotic and steroid drops, and artificial tears were used postoperatively. Chloramphenicol 0.5% was used every 4 hours for the first 10 days. Betamethasone eye drops were prescribed for every 2 hours after the procedure and gradually tapered in the following three months.

### Statistical analysis

Statistical analysis was performed using the SPSS, version 21 (SPSS Inc., Chicago, IL). A P-value < 0.05 was considered as significant. Normality of data was tested suing the Kolmogorov–Smirnov test. The Wilcoxon rank sum test was used to compare the postoperative and preoperative values. For comparison of outcomes between the two groups, the Mann–Whitney U test was performed.

##  RESULTS

Data of 47 eyes of 47 patients were available at the end of the study; 28 patients in the MyoRing group and 19 in the MyoRing + CXL group. The two study groups were matched before treatment in terms of age, UDVA, CDVA, pachymetry, keratometry (Km), and higher order aberrations. Table 1 shows the preoperative values.

**Table 1:** Mean of the preoperative data

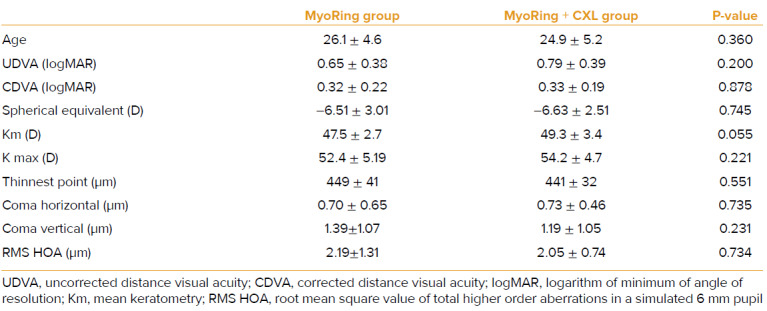

**Table 2:** Change in variables in the MyoRing group after 10 months of follow-up compared to baseline


	**Preoperative data**	**Postoperative 10- month data**	**P-value**
UDVA (logMAR)	0.65 ± 0.38	0.62 ± 0.23	0.710
CDVA (logMAR)	0.32 ± 0.22	0.33 ± 0.17	0.906
Spherical equivalent (D)	–6.51 ± 3.07	–1.8 ± 2.11	0.001
K max (D)	52.4 ± 5.19	51.5 ± 4.1	0.312
Thinnest point (µm)	449 ± 41	452 ± 40	0.423
Coma horizontal (µm)	0.70 ± 0.65	0.99 ± 0.6	0.123
Coma vertical (µm)	1.39 ± 1.08	2.01 ± 1.11	0.021
RMS HOA (µm)	2.19 ± 1.31	2.91 ± 1.23	0.006

**Table 3:** Change in variables in the MyoRing + CXL group after 10 months of follow-up compared to baseline


	**Preoperative data**	**Postoperative 10- month data**	**P-value**
UDVA (logMAR)	0.79 ± 0.39	0.52 ± 0.31	0.034
CDVA (logMAR)	0.33 ± 0.19	0.25 ± 0.16	0.118
Spherical equivalent (D)	–6.63 ± 2.51	–1.70 ± 2.41	0.001
K max (D)	54.2 ± 4.71	52.1 ± 3.75	0.032
Thinnest point (µm)	442 ± 39	445 ± 51	0.623
Coma horizontal (µm)	0.73 ± 0.46	0.37 ± 0.31	0.033
Coma vertical (µm)	1.91 ± 1.14	1.62 ± 1.42	0.081
RMS HOA (µm)	2.05 ± 0.74	2.06 ± 0.18	0.878
	
	

**Table 4:** Comparison of parameters in the MyoRing and MyoRing + CXL groups after 10 months


	**MyoRing**	**MyoRing + CXL**	**P-value**
UDVA (logMAR)	0.62 ± 0.23	0.52 ± 0.31	0.174
CDVA (logMAR)	0.33 ± 0.17	0.25 ± 0.16	0.105
Spherical equivalent (D)	–1.8 ± 2.11	–1.70 ± 2.41	0.920
Km (D)	43.8 ± 3.2	45.1 ± 3.0	0.137
K max (D)	51.5 ± 4.1	52.1 ± 3.75	0.346
Thinnest point (µm)	452 ± 40	445 ± 51	0.688
Coma horizontal (µm)	0.99 ± 0.6	0.37 ± 0.31	0.022
Coma vertical (µm)	2.01 ± 1.11	1.62 ± 1.42	0.661
RMS HOA (µm)	2.91 ± 1.23	2.06 ± 0.18	0.896
	
	

### Visual Outcomes

After 10 months, the mean UDVA improved from 0.79 ± 0.39 logMAR to 0.52 ± 0.31 logMAR (P < 0.05) in the MyoRing + CXL group. However, such an improvement was not observed in the MyoRing group. The improvement in CDVA was statistically insignificant in both groups at the end of follow-up. The increase in UDVA was observed after six months in the MyoRing + CXL group.

### Refractive Outcomes

Statistical analysis revealed that the spherical equivalent (SE) improved from –6.51 ± 3 to –1.80 ± 2 in the MyoRing group (P < 0.001) after 10 months. In the MyoRing + CXL group, the SE improved from –6.63 ± 2.5 to –1.7 ± 2 (P < 0.001). There was a significant improvement in Kmax in the MyoRing + CXL group (P < 0.05). The mean Km improved significantly in both groups (P < 0.001).

### Corneal Aberrations

The mean root mean square (RMS) total decreased by 0.55 (P > 0.1) in the MyoRing + CXL group, while it increased by 0.95 µm in the MyoRing group (P > 0.05). The mean RMS of higher order aberrations (RMS HOA) increased by 0.01 in the MyoRing + CXL group (P > 0.1) and by 0.71 µm in the MyoRing group (P < 0.05). We also observed that the horizontal coma decreased by 0.37 µm in the MyoRing + CXL group (P < 0.05). Tables 2 and 3 show the visual and topographic outcomes in the two groups after 10 months.

### Comparison of MyoRing Insertion and MyoRing Insertion + CXL

After 10 months, no significant difference was observed in the UDVA, CDVA, and Kmax in patients who underwent MyoRing insertion alone and those who underwent MyoRing + CXL. However, horizontal coma was significantly lower in the group that underwent MyoRing insertion associated with CXL. Table 4 compares the refractive and visual outcomes in both groups after 10 months.

### Complications

No complications such as migration of the MyoRing into the anterior chamber were observed during the surgery. As stated earlier, the procedure was uneventful in all of the eyes, and sutures were not needed. All eyes showed excellent tolerance to the implanted MyoRings, and no migration or extrusion was observed. Moreover, none of the eyes developed corneal ulcers or stromal necrosis superficial to the segment.

##  DISCUSSION

This study compared the refractive and corneal aberrometric effect of MyoRing insertion alone and MyoRing with concurrent CXL. As shown in the results, combining CXL with MyoRing implantation is an efficient method for keratoconus treatment and can significantly decrease the Km values. It can significantly increase the UDVA and decrease the SE. When compared to the MyoRing group, horizontal coma was significantly lower in the MyoRing + CXL group; however, there was no significant difference in other topographic and refractive outcomes.

The main indication of CXL is to halt the progression of ectatic corneal disorders such as keratoconus, pellucid marginal degeneration, and post-laser in-situ keratomileusis (LASIK) corneal ectasia.^[14,18,19]^ Collagen fibrils are crucial for corneal stability. It has been demonstrated that the diagonal links between collagen fibrils are significantly reduced in keratoconic corneas.^[20]^ This leads to corneal thinning in central and paracentral areas and causes myopia, irregular astigmatism, and decreased visual acuity. CXL stabilizes the diseased cornea by creating covalent bonds (cross links) between the collagen fibrils. It stops the progression of keratoconus without considerably changing the shape of the cornea. MyoRing implantation, on the other hand, can treat keratoconus by inducing a flattening effect, without affecting the underlying pathophysiology of ectasia. Therefore, combining CXL with MyoRing implantation would hypothetically stabilize the progression of keratoconus and improve the visual acuity by flattening the cornea. There are limited studies evaluating the outcome of MyoRing implantation with simultaneous CXL.

CXL, which has been used for treating keratoconus since 1990s, is believed to be the only treatment that can halt the progression of keratoconus. In this procedure, UVA light is irradiated to the cornea after treatment with riboflavin solution, resulting in formation of free radicals and inducing covalent bonds between amino groups of the collagen molecule, thus increasing the biomechanical stability of the cornea.^[21,22]^ In this study, we used a rather new technique for CXL; riboflavin 0.1% solution was injected into the corneal pocket created by the femtosecond laser, followed by exposing the cornea to UVA light for 10 min. The corneal epithelium is usually removed before the UV light is irradiated to the cornea to allow adequate penetration of riboflavin into the corneal stroma. Simultaneous introduction of riboflavin 0.1% into the corneal pocket created by the femtosecond laser obviates the need for epithelial debridement and reduces pain and discomfort in the early postoperative period.^[23,24]^ However, the corneal stroma composed of tightly compacted collagen fibers is fairly resistant to molecular transport. The stromal barrier can be eliminated by injecting the riboflavin solution directly into the pocket, allowing more uniform distribution in the corneal stroma. Dextran was not present in the solution as corneal toxicity might occur when it is directly injected into the corneal stroma. It has been demonstrated that a cross-linked cornea is less clear compared to a virgin cornea. Therefore, femtosecond laser penetration into the corneal stroma may be less effective in eyes that underwent CXL. El Raggal found that channel creation by femtosecond laser is more challenging in eyes that had undergone CXL. They attributed this effect to the possible rigidity of collagen fibers in the deep corneal stroma in patients treated by CXL.^[25]^ Femtosecond laser energy can be increased to overcome this problem; however, this solution may lead to a persistent corneal reaction, postoperatively.^[25]^

The keratoconic cornea is a highly aberrated cornea with significantly more coma and coma-like aberrations compared to the normal eye. In this study, horizontal coma was significantly lower in eyes that underwent MyoRing implantation with simultaneous CXL. Thus, combining CXL with MyoRing implantation might enhance the flattening effect of the segment without inducing considerable HOA. Furthermore, a synergistic effect might also be present, when CXL is combined with ICRS implantation, augmenting the therapeutic effect that is observed with either treatment.^[15]^

Hafez recently studied the outcomes of KeraRing and MyoRing implantation with simultaneous CXL.^[15]^ Patients who underwent MyoRing implantation plus CXL showed a significant reduction in Kmean. However, there was little improvement in the astigmatic component of keratoconus with MyoRing implantation, and significant reduction of astigmatism was observed in patients treated by combination of KeraRing and CXL.

In a retrospective study by Bikbova et al, the efficacy of MyoRing implantation and MyoRing implantation + CXL after three years was reported. They found that MyoRing implantation with simultaneous CXL had slightly better outcomes, although the effect of MyoRing implantation alone was stable over time.^[26]^ Similarly, in this study, we found that the topographic and visual outcomes were comparable in the two groups after 10 months, although horizontal coma was significantly lower in patients who underwent MyoRing implantation with simultaneous CXL.

El Raggal evaluated the outcome of combined KeraRing insertion with CXL, performed in a single session or with a six-month interval. He observed that there was a significant improvement in UDVA, CDVA, and keratometric values. However, patients treated using the same-day method had better topographic outcomes.^[27]^

Coskunseven and colleagues found that ICRS implantation followed by CXL is more effective in improving CDVA, SE, and the mean Km in keratoconus compared to CXL followed by ICRS implantation. The mean interval between the treatments was seven months.^[28]^

None of the patients during the study developed segment decentration and extrusion, partly due to application of femtosecond laser, which creates a more precise depth of incision.

Despite the limited number of cases, this study shows that the MyoRing + CXL procedure is a safe and effective method for treating keratoconus. It is comparable to MyoRing insertion alone for improvement in visual and topographic outcomes in the short term. Moreover, a significant reduction in HOA is observed with the MyoRing + CXL procedure, which is of paramount importance in highly aberrant corneas with keratoconus. More studies with a longer follow-up are needed to better elucidate the efficacy and safety of this procedure.

##  Financial Support and Sponsorship

Nil.

##  Conflicts of Interest

There are no conflicts of interest.
